# Myocardial fibrosis in the posterior myocardium in Fabry disease is associated with global rather than regional longitudinal strain reduction

**DOI:** 10.1093/ehjci/jeae279

**Published:** 2024-10-29

**Authors:** Niccolo’ Maurizi, Guillaume Barbey, Alessandra Pia Porretta, Sarah Hugelshofer, Dimitri Arangalage, Panagiotis Antiochos, Juerg Schwitter, Frédéric Barbey, Pierre Monney

**Affiliations:** Cardiology, Lausanne University Hospital (CHUV), Rue du Bugnon 46, Lausanne 1011, Switzerland; Cardiology, Lausanne University Hospital (CHUV), Rue du Bugnon 46, Lausanne 1011, Switzerland; Cardiology, Lausanne University Hospital (CHUV), Rue du Bugnon 46, Lausanne 1011, Switzerland; CNMR Maladies Cardiaques Héréditaires Rares, APHP, Hôpital Bichat, Paris, France; Cardiology, Lausanne University Hospital (CHUV), Rue du Bugnon 46, Lausanne 1011, Switzerland; Cardiology, Lausanne University Hospital (CHUV), Rue du Bugnon 46, Lausanne 1011, Switzerland; Cardiology, Lausanne University Hospital (CHUV), Rue du Bugnon 46, Lausanne 1011, Switzerland; University of Lausanne (UNIL), Lausanne 1011, Switzerland; Cardiology, Lausanne University Hospital (CHUV), Rue du Bugnon 46, Lausanne 1011, Switzerland; University of Lausanne (UNIL), Lausanne 1011, Switzerland; University of Lausanne (UNIL), Lausanne 1011, Switzerland; Immunology and Allergy, Lausanne University Hospital (CHUV), Lausanne, Switzerland; Cardiology, Lausanne University Hospital (CHUV), Rue du Bugnon 46, Lausanne 1011, Switzerland; University of Lausanne (UNIL), Lausanne 1011, Switzerland

Detecting cardiac involvement and myocardial fibrosis in Fabry disease (FD) is crucial because therapy introduction in the initial disease phases is more likely to alter progression.^1^ Myocardial fibrosis may precede left ventricular hypertrophy (LVH) in females, but it usually indicates a later stage of FD, associated with a poor treatment response.^2^ In FD, myocardial fibrosis is specifically located in the infero-lateral wall and can easily be detected by cardiovascular magnetic resonance (CMR) using late gadolinium enhancement (LGE). However, frequent CMR follow-up can be impractical, and early echocardiographic markers of myocardial involvement can be clinically relevant. Early mechanical dysfunction detected by global longitudinal strain (GLS) has been correlated with initial myocardial involvement, even in the absence of LGE,^3^ and some authors suggested that strain reduction preferentially affects the infero-lateral wall, mirroring the distribution of myocardial fibrosis.^4^ Whether GLS effectively tracks disease progression and how it correlates with its severity have only been partially investigated. We aimed to explore the relationship between GLS and myocardial fibrosis by CMR, to understand whether a specific pattern of GLS may reflect the presence of fibrosis in patients with FD cardiomyopathy.

A total of 28 genetically proven FD patients (42 ± 15 years, 46% hemizygous males) who underwent both echocardiography with GLS and CMR during the same year were included. GLS analysis was performed offline on an EchoPAC workstation (GE Healthcare, v113.04) from three dedicated apical views of the LV acquired with a frame rate > 50 images/s. Myocardial fibrosis was assessed by late LGE, quantified as a percent of the LV mass (6 SD method).

Twenty-two patients (60% males, 46 ± 18 years) were on enzyme replacement treatment for 5 [2;9] years, and 18 (64%, 8 males) presented with LVH. Mean LV ejection fraction was 66 ± 4%, whereas left atrial indexed volume was 27 ± 9 m mL/m^2^. The mitral E/A ratio and average E/e′ ratio were 1.4 ± 0.4 and 10.2 (6.3–13.4), respectively. LGE was found in 8 (29%), detected in the lateral and infero-lateral walls, with a mean volume of fibrosis of 2.1% (1.3–3.2) of the myocardium. The LGE-positive subgroup was characterized by a higher prevalence of LVH (88% vs. 5%, *P* < 0.001), was older (57 ± 9 vs. 36 ± 13 years, *P* < 0.01), and had lower renal function [estimated Glomerular Filtration Rate (eGFR) 79 ± 24 vs. 106 ± 19 mL/min, *P* < 0.01] and a higher diastolic E/e′ ratio [13.5 (10.5–23.0) vs. 6.7 (5.4–8.0), *P* < 0.001).

GLS (absolute value) was significantly lower in LGE-positive patients [13.3 (12.6–15.6) vs. 19.2% (18.3–21.4), *P* < 0.001). Importantly, GLS reduction was observed in all the LV walls and never restricted to the LGE-positive walls (*Figure 1*). Specifically, among patients with LGE detected in the lateral region, longitudinal strain of the lateral region was not lower compared with the anterior (13.3 ± 3.0 vs. 14.1 ± 2.5%, *P* = 0.22) or the inferior region (13.3 ± 3.0 vs. 12.9 ± 3.5%, *P* = 0.73). The longitudinal strain was highest in the apical, lower in the mid-ventricular, and lowest in the basal region. Reduction of mean GLS was directly proportional to the quantity of fibrosis: 19.2 (18.3–21.4) in those without LGE, 14.2 (12.8–15.2) in those with 1 segment LGE, and 12.8 (11.2–13.4) in those with >1 segment LGE (*P* for trend < 0.01) (*Figure 1*). By receiver operating characteristics (ROC) analysis, a GLS < 19.9% could detect the presence of LGE, LVH, or concentric remodelling with a sensitivity of 86% and a specificity of 71%; at a cut-off value of GLS < 18.1%, the sensitivity and specificity were 57% and 100%, respectively. Bivariate logistic regression identified age [odds ratio (OR) 1.29, 95% confidence interval (CI) 1.00–1.65, *P* = 0.04), LV mass index (OR 1.10, 95% CI 1.02–1.18, *P* = 0.01), eGFR (OR 0.92, 95% CI 0.86–0.97, *P* = 0.02), and GLS (OR 0.44, 95% CI 0.25–0.78, *P* < 0.01) as significantly associated with LGE.

**Figure 1:**
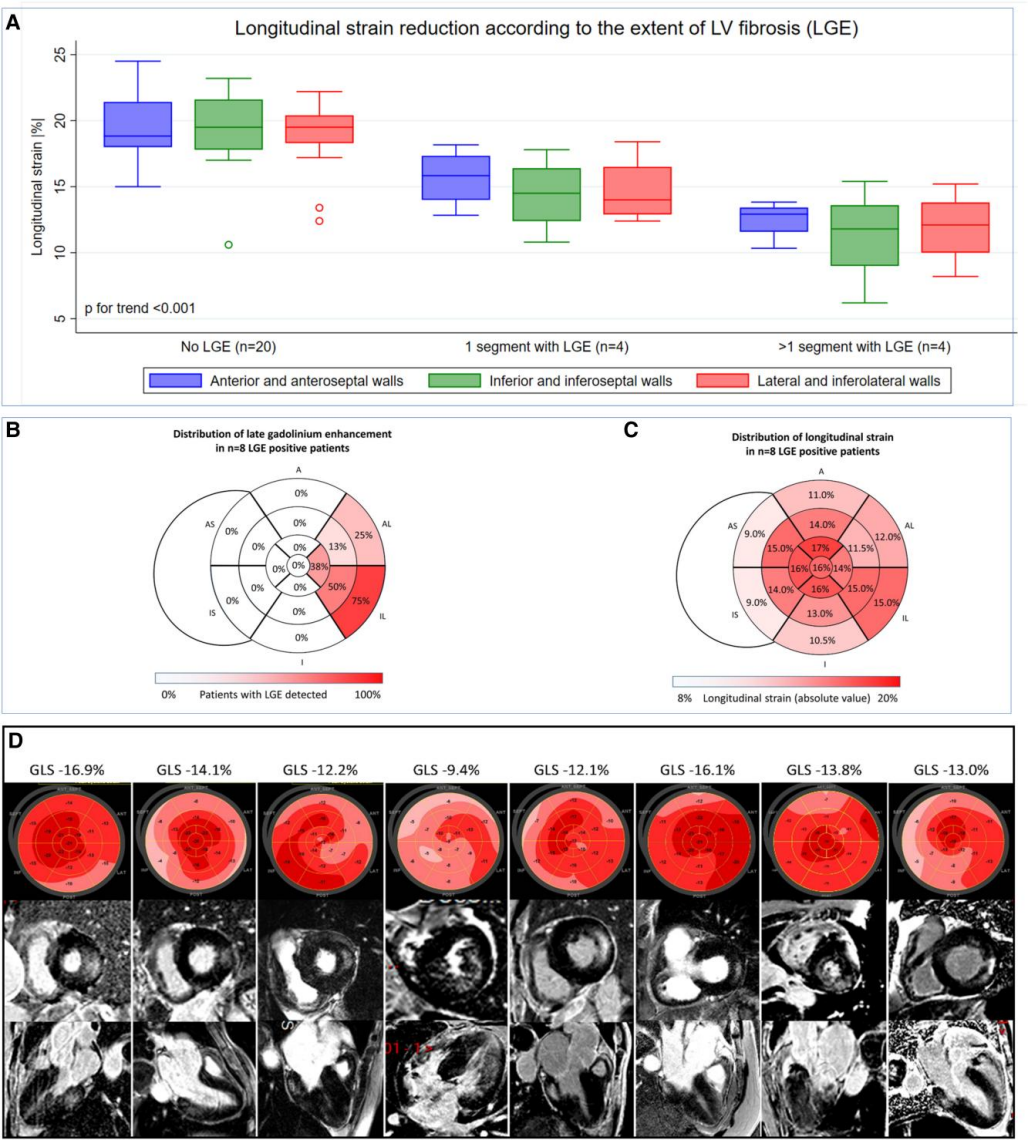
Comparison of global and segmental longitudinal strain reduction according to the extent and distribution of myocardial fibrosis in Fabry cardiomyopathy. (*A*) The GLS of the anterior, inferior, and lateral regions in relation to the presence and quantity of LGE at cardiac magnetic resonance. (*B*) The bull’s eye distribution of the LGE is presented. (*C*) The bull’s eye depicting the average strain values is displayed. (*D*) The longitudinal strain of bull’s eye maps (top row) and the LGE images in basal short-axis orientation (mid-row) and three-chamber long-axis orientation (lower row) for each individual patient with FD complicated by myocardial fibrosis. LGE, late gadolinium enhancement; GLS, global longitudinal strain; M, male; F, female.

Timely identification of potential progressors to more severe cardiac phenotypes in FD is of pivotal clinical relevance.^5^ The present study showed several actionable messages for the practising cardiologist: (i) GLS decreased with increasing severity of cardiac involvement; (ii) its reduction was more important in the basal segments compared with the mid-ventricular and apical segments; and (iii) in patients with LGE of the posterolateral myocardium, the reduction in longitudinal strain was relatively homogenously distributed across the LV walls and not restricted to or worse in the posterolateral region affected by fibrosis. Krämer *et al*.^4^ suggested that a segmental longitudinal strain reduction in the basal infero-lateral wall was associated with the presence of fibrosis in the same distribution, a concept challenged by our observations. Global rather than local strain reduction is consistent with the pathophysiological hypothesis that sphingolipid storage is a global phenomenon and that the degree of deposition directly correlates with the decrease in myocardial deformation. Finally, we observed that already mild reduction in GLS (typically between 18 and 20%) should be considered as potentially abnormal and prompt the realization of a CMR scan as it was associated with the composite endpoint of LVH, concentric remodelling or LGE. Such strategy may carry crucial clinical implications for patients affected by the cardiac variant form of FD.

## Data Availability

Patients did not consent for further transfer of their data. Ethics committee protocol is CER-VD 2021-00440/2024-01420.
